# Supplementary material for: A Critical View of the Application of the APEX Software (Aqueous Photochemistry of Environmentally-Occurring Xenobiotics) to Predict Photoreaction Kinetics in Surface Freshwaters
Source: Molecules. 2019 Dec 18;25(1):9. doi: 10.3390/molecules25010009 (PMC7017383; doi:10.3390/molecules25010009)
Supplement: Supplementary file 1 [file molecules-25-00009-s001.zip › 3.2.4_gcc-4.4.0.zip!/3.2.4_gcc-4.4.0/3.2.4_gcc-4.4.0/doc/octave/html/interpreter/Character-Strings-in-Mex_002dFiles.html]

Character Strings in Mex-Files - Untitled


Next: Cell Arrays with Mex-Files,
Previous: Working with Matrices and Arrays in Mex-Files,
Up: Mex-Files


---

#### A.2.3 Character Strings in Mex-Files

As mex-files do not make the distinction between single and double
quoted strings within Octave, there is perhaps less complexity in the
use of strings and character matrices in mex-files. An example of their
use, that parallels the demo in stringdemo.cc, is given in the
file mystring.c, as seen below.

```
```
     #include <string.h>
     #include "mex.h"
     
     void
     mexFunction (int nlhs, mxArray *plhs[], int nrhs, 
     	     const mxArray *prhs[])
     {
       mwIndex i, j;
       mwSize m, n;
       mxChar *pi, *po;
     
       if (nrhs != 1 || ! mxIsChar (prhs[0]) || 
           mxGetNumberOfDimensions (prhs[0]) > 2)
         mexErrMsgTxt ("expecting char matrix");
     
       m = mxGetM (prhs[0]);
       n = mxGetN (prhs[0]);
       pi = mxGetChars (prhs[0]);
       plhs[0] = mxCreateNumericMatrix (m, n, mxCHAR_CLASS, 
     				   mxREAL);
       po = mxGetChars (plhs[0]);
     
       for (j = 0; j < n; j++)
         for (i = 0; i < m; i++)
           po [j*m + m - 1 - i] = pi [j*m + i];
     }
```
```

An example of its expected output is

```
     mystring(["First String"; "Second String"])
     => s1 = Second String
             First String
```

Other functions in the mex interface for handling character strings are
`mxCreateString`, `mxArrayToString`, and
`mxCreateCharMatrixFromStrings`. In a mex-file, a character string
is considered to be a vector rather than a matrix. This is perhaps an
arbitrary distinction as the data in the mxArray for the matrix is
consecutive in any case.
